# P10 Biomarker Driven Antifungal Stewardship in Acute Leukaemia (BioDriveAFS)—a multicentre randomized controlled trial to assess clinical and cost effectiveness

**DOI:** 10.1093/jacamr/dlac053.010

**Published:** 2022-05-31

**Authors:** Thomas Taynton, Adwoa Parker, Lydia Flett, Joanne Laycock, Joanne Newman, Caroline Fairhurst, William Hope, Patrick Lillie, Puvanendran Tharmanathan, Belén Corbacho, Laura Sheard, Andrea Hilton, Shreyans Gandhi, Hannah Gray, Tracey Guise, Jacqui Sneddon, David Torgerson, David Allsup, Gavin Barlow

**Affiliations:** 1 Hull York Medical School, Hull University Teaching Hospitals NHS Trust, Hull, UK; 2 York Trials Unit, York, UK; 3 University of Liverpool, Liverpool, UK; 4 Hull University Teaching Hospitals NHS Trust, Hull, UK; 5 University of Hull, Hull, UK; 6 Kings College Hospital NHS Foundation Trust, UK; 7 University of York, York, UK; 8 BSAC, Birmingham, UK

## Abstract

**Background:**

The BioDriveAFS trial aims to investigate whether a biomarker-based antifungal stewardship strategy is superior to a prophylactic antifungal strategy, including existing standard of care, in reducing antifungal therapy use in patients with acute leukaemia, without impacting health-related quality of life at 12 months. The trial will be commencing in the UK in 2022 and aims to recruit 500 patients across 40 sites. We hypothesized that a biomarker-led monitoring approach would be non-inferior to the standard of care approach utilizing antifungal prophylaxis.

**Patients and methods:**

Patients diagnosed with acute leukaemia who are planned to have intensive chemotherapy will be randomly allocated to one of two arms. The biomarker arm will consist of twice-weekly galactomannan and β-d-glucan until the end of intensive chemotherapy; positive biomarker results, neutropenic fever non-responsive to broad-spectrum antibacterials, or clinical suspicion will lead to investigation for potential invasive fungal infection as per international guidelines. Patients with proven or probable invasive fungal infection (IFI), as per the consensus definitions, will receive therapeutic antifungals, whereas those with possible or no IFI will have antifungals withheld. The control arm consists of local standard-of-care antifungal prophylaxis, including mould-active, without regular biomarker monitoring.

**Results:**

Primary outcome measures are exposure to therapeutic antifungal therapy and patient quality of life at 12 months versus baseline. Secondary outcome measures include total antifungal exposure, adverse events and complications, proven and probable IFI and treatment outcome, overall survival, all-cause mortality and IFI-related mortality. Resource use to determine cost-effectiveness and antifungal resistance in fungi will also be measured.

**Funding:**

The trial is funded by the National Institute for Health Research Health Technological Assessment Programme (NIHR132674) and supported by BSAC.

